# Effective number of different populations: A new concept and how to use it

**DOI:** 10.1002/ece3.70303

**Published:** 2024-09-15

**Authors:** Evsey Kosman, Frida Feijen, Jukka Jokela

**Affiliations:** ^1^ Institute for Cereal Crops Research, School of Plant Sciences and Food Security, The George S. Wise Faculty of Life Sciences Tel Aviv University Tel Aviv Israel; ^2^ ETH Zurich, Department of Environmental Systems Science Institute of Integrative Biology (IBZ) Zürich Switzerland; ^3^ EAWAG Aquatic Ecology Dübendorf Switzerland

**Keywords:** assignment‐based distance, *Atriophallophorus winterbourni*, differentiation, dispersion, diversity, Hill numbers, β‐variation

## Abstract

Widely used methods to assess population genetic structure and differentiation rely on independence of marker loci. Following the assumption, the common metrics, for example *F*_ST_, evaluate genetic structure by averaging across loci. Common metrics do not use information in the associations among loci at the individual level and are often criticized for failing to measure true differentiation even when loci segregate independently. We introduce a new concept to measure β‐variation (Effective Number of Different Populations, ENDP). It requires the following steps: (a) calculation of a proper dissimilarity between genetic profiles of all individuals; (b) calculation of suitable pairwise distances between the samples based on the dissimilarities between individuals; (c) calculation of diversity (in terms of Hill numbers) and dispersion of samples based on the pairwise distances between samples; (d) ENDP is then estimated by combining the diversity and dispersion. ENDP estimates β‐variation independently of estimates of within‐sample α‐variation, although β‐ and α‐estimates could statistically correlate to some extent. This new concept differs from the existing standard where β‐diversity is estimated based on the “partition of variation” scheme (beta=gamma−alpha or beta=gamma/alpha), so that estimates of β‐diversity directly depend on the corresponding values of α‐diversity. ENDP estimates are obtained by evaluating information in the available genetic profiles of individuals including association of loci. Therefore, ENDP can be used even in an absence of panmixia. We illustrate the use of this concept by analyzing the population genetic structure of a sexual species (a trematode parasite) occupying connected populations across a broad geographic area. The analysis is complicated by geographically coexisting cryptic species and the potential mixed‐mating system of this hermaphroditic parasite. Analyses with subsampled data demonstrated that ENDP estimates are robust. Number of loci used for genotyping has much stronger effect on variation of point ENDP estimates than sample size.

## Graphical abstract

A new concept to measure β‐variation (Effective Number of Different Populations, ENDP) is introduced. Estimates of ENDP are obtained by evaluating information in the available genetic profiles of individuals including association of loci. The use of this concept is illustrated by analyzing the population genetic structure of a sexual species (a trematode parasite) occupying connected populations across a broad geographic area.

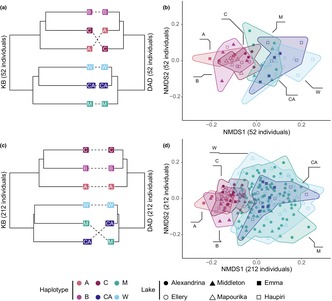

## 1 INTRODUCTION

Discovering the genetic structure of populations is one of the key applications of population genetic markers. Not surprisingly, methods aimed at assessing the extent of difference among subdivided populations are numerous and have nearly always been central part of the standard population genetics toolkits. Historically, the first $F_{\mathrm{ST}}$ measure (Wright, 1951), as its many later analogues, aimed at understanding the divergence of populations in relation to evolutionary processes (Excoffier et al., 1992; Nei, 1973; Slatkin, 1995). Later, one of the specific applications has been to estimate the partitioning of genetic variation within and among subdivided populations (Hedrick, 2005; Meirmans & Hedrick, 2011; Nei & Chesser, 1983). A comprehensive review of methods aimed at differentiation of molecular diversity with an emphasis on information (entropy) analysis can be found in Sherwin et al. (2017).

The $F_{\mathrm{ST}}$ measures were developed for single loci. Many of the commonly used multilocus estimates evaluate each locus independently with further averaging across loci ignoring information that is in the associations between loci (i.e., multilocus genotypes) and between alleles within a diploid (or polypoid) locus. Thus, $F_{\mathrm{ST}}$ and its relatives ($G_{\mathrm{ST}}$, $G_{\mathrm{ST}}^{\prime }$, $G_{\mathrm{ST}}^{\prime \prime }$, $\varphi_{\mathrm{ST}}$, $R_{\mathrm{ST}}$) are not sensitive to divergence among populations that exist only due to differences in association of alleles and/or loci (allele frequencies are equal in all populations). For example, two populations *P*_1_ and *P*_2_ consisting of individuals with different binary genotypes at four loci (1010 and 0101 in *P*_1_, and 1111, 1100, 0011 and 0000 in *P*_2_) are indistinguishable with $F_{\mathrm{ST}}$ and its relatives, if frequencies of each binary allele 1 and 0 are equal in *P*_1_ and *P*_2_ (e.g. *P*_1_ and *P*_2_ consist of four individuals each with the above‐mentioned genotypes: $P_{1}=\left\{1010,1010,0101,0101\right\}$ and $P_{2}=\left\{1111,1100,0011,0000\right\}$; frequencies of all binary alleles equal 0.5).

The classical approach works best in a fully recombining panmictic sexual population. However, the classical approach may work less well in populations with clonal reproduction, a mixed mating system or where unknown cryptic species coexist. To measure the variation and divergence of such populations, new metrics that use information based on associations of loci have emerged during the last decades (Gillet et al., 2004; Gregorius et al., 2003; Kosman, 1996, 2014; Kosman & Leonard, 2007). These metrics also include measures aimed at evaluating the extent and significance of differences among populations (Czajowski et al., 2021; Gillet, 2013; Gillet & Gregorius, 2008; Gregorius, 2010; Gultyaeva et al., 2020; Kosman et al., 2014).

Jost (2008) criticized shortcomings of the standard metrics that are commonly called measures of “differentiation” ($F_{\mathrm{ST}}$, $G_{\mathrm{ST}}$, $\varphi_{\mathrm{ST}}$, $R_{\mathrm{ST}}$) because they can provide unrealistic estimates of the differences in the structure of the populations, especially if the within‐population variation is very high. Therefore, using the term “differentiation” for those measures seems inappropriate and confusing. Second, these estimates are unintuitive and can even be misleading (see Jost, 2008). To be more specific, it is possible that these measures do not reach their maximum values, could be far away from maximum and approach zero (indication of no differentiation), even for populations that do not share any alleles. The latter problem was resolved to some extent by $G_{\mathrm{ST}}^{\prime }$ and $G_{\mathrm{ST}}^{\prime \prime }$ metrics (Hedrick, 2005; Meirmans & Hedrick, 2011), and solved for a separate locus with a metric introduced by Jost (2008): measure of differentiation D that reaches its maximum 1 when differentiation is complete. Nevertheless, new ideas are still needed for finding an intuitively acceptable approaches to measuring variation among populations especially in a case of multilocus genotypes.

Variation within a population (below we refer to population as “OU,” that is, Operational Unit) could be thought of and described in different ways. There are two major facets of variation—diversity and dispersion (Gregorius & Gillet, 2015). Diversity is about individual types within a given OU, when all nonidentical types are considered equally distant, while dispersion is about an overall relationship between individual types based on pairwise dissimilarities between them. These attributes of variation are independent in the sense that OUs can be equally diverse for a wide range of dispersion estimates, and values of dispersion can vary from extremely small to extremely large for highly diverse OUs. However, when diversity is low, dispersion estimates are also small, whereas high dispersion estimates predetermine large values of diversity.

Differentiation is a common but ambiguously used term. In a general context, differentiation is about the overall relationship among several OUs considered together as a group (e.g., a metapopulation defined as *a group of populations*) and refers to how a total variation of that group can be partitioned among and within those OUs. Classical measures of “differentiation” ($F_{\mathrm{ST}}$, $G_{\mathrm{ST}}$, $\varphi_{\mathrm{ST}}$, $R_{\mathrm{ST}}$) are based on assessment of the extent to which variation of individuals within the group of OUs (e.g., all individuals of metapopulation) exceeds the corresponding average variation within each constituent OU. However, as we pointed out above, when diversity within each OU is very high (e.g. large number of equally frequent alleles), such “differentiation” measures are counterintuitive because they deliver very small scores even when OUs are completely different (e.g., populations share no alleles). Therefore, we would not recommend using the term “differentiation” in such a general context and suggest replacing it by “structural variation” among OUs. We propose to use the term “differentiation” for a much more specific context (see below) requesting that estimates of “true” differentiation must increase with (i) a rise of an overall difference between OUs (dispersion of OUs), and (ii) a higher regularity of distribution of pairwise differences between OUs (diversity of OUs), provided that all other characteristics of relationships among OUs being identical.

The measures of biological variation proposed in this paper combine the diversity and dispersion perspectives with the diversity component being conceptually similar to metrics developed by Hill (1973) and Jost (2007, 2008) advocating the use of numbers equivalents for estimating diversity. Such measures can be used, for example, to conclude and compare the effective numbers of different species within a community, or effective number of different communities within a landscape. According to Jost (2008), the properties of the corresponding diversity measures, when applied to alleles of genotypes, satisfy the expectations for answering population genetic questions in providing intuitively correct answers to a series of practical and theoretical questions. The main idea of Hill's approach is the multiplicative nature of diversity partitioning.

$$\left(\text{total diversity}\right)=\left(\text{diversity within subunits}\right)\times \left(\text{diversity among subunits}\right)$$

which allows independent estimates of within‐ and among‐subunit components (Jost, 2007, 2008). In other words, the effective number of alleles, genotypes, or any chosen attribute in a set of OUs equals the product of the corresponding effective number per OU and the effective number of distinct OUs. Such diversity estimates are intuitive, easy to interpret and can be used in various applications (e.g., for management of populations and in conservation biology). The effective number of distinct populations is an absolute measure of population differentiation. Based on the proportion of total diversity that is contained in the average population in terms of effective numbers, Jost (2008) introduced a new non‐negative measure of differentiation D that reaches its maximum 1 when differentiation is complete. Conceptual aspects of diversity partitioning and measuring diversity components based on the most general definition of effective numbers (Hill numbers are a partial case) were thoroughly considered by Gregorius (2016).

For multilocus genotypes, differentiation D is obtained by averaging across all loci. Then D reflects the average differentiation within separate loci in a given set of populations rather than differentiation between the populations due to differences in distribution and association of alleles among loci in multilocus genotypes. If two populations have identical allele distributions at each locus but non‐identical association of those alleles into the corresponding multilocus genotypes, then no differentiation is detected (D=0). The same shortcoming characterizes all commonly used $F_{\mathrm{ST}}$ related measures ($G_{\mathrm{ST}}$, $G_{\mathrm{ST}}^{\prime }$, $G_{\mathrm{ST}}^{\prime \prime }$, $\varphi_{\mathrm{ST}}$, $R_{\mathrm{ST}}$) that do not actually measure differentiation. Chao et al. (2015) further demonstrated that the heterozygosity‐based “differentiation” measures, such as $G_{\mathrm{ST}}$ and Jost's D, do not possess two of the essential monotonicity properties: differentiation never decrease when (i) a new unshared allele is added to a population, and (ii) when some copies of a shared allele are replaced by copies of an unshared allele. Thus, being more intuitive, Jost's “differentiation” metric D is not free of the shortcomings of the standard measures (violation of monotonicity property, inability to take into account association between loci) and may deliver inadequate estimates and even miss the actual difference between populations.

Nearly all papers cited above and many others (Heller & Siegismund, 2009; Ryman & Leimar, 2009) debate the pros and cons of a variety of “differentiation” measures considering numerous critical examples. A part of the problem is that there are two different perspectives to partitioning total genetic variation—differentiation and apportionment (Gregorius, 2009, 2010, 2016; Gregorius & Gillet, 2015), although separation between them is not clearly made.

Differentiation among populations describes a tendency of the same allele or genotype to occur in the same population reporting a maximum when all populations consist of unique alleles (genotypes) (i.e., populations do not share alleles, but each population may be polymorphic for each locus). Jost *D* is assumed to be an example of a differentiation measure although it has its own shortcomings.

Apportionment, on the other hand, describes a tendency of individuals with different alleles or genotypes to occur in different populations. Maximum apportionment is reached when each population is fixed for a different allele (or genotype), that is, populations are monomorphic but have different genotypes. This means that maximum of differentiation among populations is necessary but not sufficient condition of maximum apportionment (if all genotypes are considered equally dissimilar). Thus, apportionment metrics measure the extent of fixation of distinct alleles or genotypes among populations (e.g. fixation index $F_{\mathrm{ST}}$).

There are a few immediate consequences of theoretical and practical importance for geneticists for considering the dual perspectives of differentiation and apportionment. First, $F_{\mathrm{ST}}$‐like indices (e.g., $G_{\mathrm{ST}}$, $G_{\mathrm{ST}}^{\prime }$, $G_{\mathrm{ST}}^{\prime \prime }$, $\varphi_{\mathrm{ST}}$, $R_{\mathrm{ST}}$) provide a kind of apportionment (fixation) estimates based on variance partitions, even if they are commonly declared and used as measures of differentiation among populations. Second, Jost's “differentiation” metric D (Jost, 2008) is actually closer to measuring differentiation among populations, not apportionment. This may explain, at least in part, inconsistency in some results obtained with D and the $F_{\mathrm{ST}}$ based measures. Third, valid differentiation measures can reach their maximum (absolute differentiation) independently of the degree of genetic variation within populations, that is, even if the populations are not fixed to alternative alleles or genotypes (such situation is impossible with $F_{\mathrm{ST}}$ and $G_{\mathrm{ST}}$).

In this paper, our purpose is to further expand the differentiation perspective for studies of population structure. The idea is to express diversity of populations in terms of the effective number of equally distant populations. This allows estimation of differentiation in a way that is independent of both total diversity (*γ*‐diversity) of a given metapopulation and diversity within its constituents (*α*‐diversity). Determining the effective number is based on pairwise genetic distances between populations, though only the proportional contributions of those distances to the total sum of distances are utilized. Such diversity index depends only on the relative position of populations to each other in the given genetic landscape and measures regularity of relationships among populations. Therefore, an identical value of diversity index is returned for any metapopulation consisting of the same number of populations, even if all pairwise genetic distances (magnitudes of genetic differences) change proportionally (e.g., for two sets of three populations with relationships among the populations represented geometrically by two similar shaped but different size triangles). For example, if each of three populations is fixed to a single binary genotype at six loci in two metapopulations $A=\left\{\left(100000\right),\left(001000\right),\left(000010\right)\right\}$ and $B=\left\{\left(110000\right),\left(001100\right),\left(000011\right)\right\}$, then *A* and *B* are of identical diversity among their constituent populations, although pairwise genetic differences between the three populations in *A* are two times smaller than those in *B*.

To distinguish between two different metapopulations with the same diversity (as measured in terms of effective number of equally distant populations), the diversity concept must be integrated with the dispersion concept. The dispersion component of variability is expressed in terms of genetic distances between populations. Combined metrics of diversity and dispersion components will be then called the Effective Number of Different Populations (ENDP). Such metrics are completely predetermined by pairwise genetic distances between populations, their magnitudes and regularity of distribution, and deliver exhaustive estimates of variation among populations within the corresponding metapopulation. Basic principles of our approach are similar to those developed by Scheiner et al. (2017) for ecological communities [Gregorius and Kosman (2018) considered a more general case of integration of the diversity and dispersion concepts].

We test the relevance of the suggested metrics with two empirical data sets. First, we use data published by Feijen et al. (2022) describing population and species structure of the New Zealand trematode parasite species in the genus *Atriophallophorus* spp. using nuclear SNP markers and mitochondrial haplotypes based on a part of the NADH5 gene. This parasite uses the snail *Potamopyrgus antipodarum* as its intermediate host and waterfowl as the definitive host. The parasite has a sexual stage in the definitive host while the reproduction in the snail host is clonal. Feijen et al. (2022) found support for cryptic species structure in the parasite populations by applying computationally demanding multispecies coalescent models on a subset of individual parasites (*N* = 52) [Bayes Factor Delimitation (Leache et al., 2014)]. They further used regression analyses on pairwise genetic distances among individuals (*N* = 462). Both analyses supported the conclusion that the samples represent at least two distinct species that coexist in broad geographic range (see figure 2 in Feijen et al., 2022). Here we use the same subset of genotypes and the full set of genotypes as in the two analysis by Feijen et al. (2022) to calculate both the effective number of equally distant populations and the ENDP in samples that are known to represent two coexisting cryptic species.

Second, we applied the new metric to assess population genetic structure of the common species, *Atriophallophorus winterbourni*. We asked what the effective number of equally distant and different populations is in these locations which cover the geographic regions of South Island of New Zealand. We contrast our results to a more detailed analysis of connectedness of these populations presented in Feijen et al. (2022).

We use these data to raise the question whether it would be reasonable to incorporate estimates of ENDP into analyses aiming to understand diversity and structure of populations using genetic markers. An important reason for selection of those data was the fact that they were already analyzed with other state‐of‐the‐art tools that allow a direct and effective comparison of the new delivered results with those reported previously. We also discuss the rationale and applicability of these metrics.

## 2 MATERIALS AND METHODS

We develop metrics for measuring structural variation in a metapopulation based on a matrix of pairwise genetic distances between the populations. Distances between the populations are measured using the dissimilarity‐based approaches (Kosman, 2014; Kosman & Leonard, 2007) although other distances can also be applied. This approach requires a proper assessment of dissimilarity between individual genotypes.

### 2.1 Dissimilarity between individual genotypes

Choice of a suitable dissimilarity measure is a key factor for valid analysis of genetic variation. The selection depends on ploidy of a given organism and the type of molecular markers used for estimating genetic variation (Kosman & Jokela, 2019; Kosman & Leonard, 2005). Here, we use nuclear SNP polymorphism of *Atriophallophorus* spp. (Feijen et al., 2022) to examine population genetic structure. Since SNPs are codominant markers and *Atriophallophorus* spp. is a diploid organism, we calculated dissimilarity between the SNP genotypes (δ) according to equation (3) in Kosman and Leonard (2005) or equation (6) in Kosman and Jokela (2019). Here, the dissimilarity between two genotypes at one diploid locus equals 1, 0.5 and 0, if the genotypes do not share any allele, share one allele, or have identical pair of alleles, respectively. Then the average across all loci delivers dissimilarity δ between the two multilocus genotypes.

### 2.2 Distance between populations

The most used genetic distance measures between populations are based on allele frequencies, averaging independent estimates at each locus over all loci [e.g. Nei's genetic distances (Nei, 1972)]. Allele‐frequency based measures do not consider possible associations between different loci, so that two populations with no shared genotypes can be declared identical if they share the same alleles at equal frequencies. Therefore, considering associations between loci would be important for metrics of genetic distances between populations.

The two types of distances based on dissimilarities between individuals are calculated by averaging individual dissimilarities (both between and within populations) and by assignment of individuals from two populations based on their dissimilarities without the effect of dissimilarities within populations [Kosman, 2014). The average‐based approach (distance of average differences, ${\mathrm{DAD}}_{\rho }$, equation (2) in Kosman and Leonard (2007)] may have undesirable mathematical properties for some dissimilarity measures ρ as ${\mathrm{DAD}}_{\rho }$ can be negative or zero for distinct populations. For example, ${\mathrm{DAD}}_{m}$, which is the distance of average differences for the simple mismatch coefficient m, can be zero for distinct populations as it is identical to Nei's minimum genetic distance (Kosman & Leonard, 2007). Therefore, the distance of average differences does not properly work in the case of association between loci. An alternative, the assignment‐based genetic distance (KB) developed by Kosman (1996) and Gregorius et al. (2003), is a generalization of the mathematical notion of distance between two sets of scattered points (Kosman, 2014). Kosman distance (KB) can distinguish between populations where linkage of markers is variable for a same set of alleles, and it is suitable for comparison of populations with strong linkage patterns as is the case for asexual or mixed mode of reproduction, or with cryptic structure due to unidentified coexisting species.

One strength of dissimilarity‐based methods is the ability to deal with missing data. Dissimilarity between a given pair of genotypes can be calculated using all the data that are available for both individuals (only loci with missing genotypes are excluded).

We applied the dissimilarity‐based distances ${\mathrm{DAD}}_{\delta }$ and ${\mathrm{KB}}_{\delta }$ to measure genetic differences between the parasite populations *Atriophallophorus* spp. (SNP markers), where δ is dissimilarity between the multilocus SNP genotypes mentioned beforehand in the previous section. Since the mode of parasite reproduction is mixed with prevailing outcrossing, we used the ${\mathrm{DAD}}_{\delta }$ distance as the benchmark for calculations assuming that association between loci is minimal, if any. As Feijen et al. (2022) also discovered a cryptic species structure in their *Atriophallophorus* spp. samples, we also calculated effective numbers based on ${\mathrm{KB}}_{\delta }$ distances. This is to show how dissimilarity‐based distances, ${\mathrm{DAD}}_{\delta }$ and ${\mathrm{KB}}_{\delta }$, can be used to study structural variation in cases where it is not known if there are groups within‐populations that differ in their linkage structure.

### 2.3 Metrics of variation

#### 2.3.1 Diversity

We first construct metrics of variability similarly to Scheiner et al. (2017). For a set of *S* Operational Units (OUs; single populations in our analysis), let $d_{\mathrm{ij}}$ be any distance between *i*th and *j*th OUs ($0\leq d_{\mathrm{ij}}\leq 1,d_{\mathrm{ij}}=d_{\mathrm{ji}},d_{\mathrm{ii}}=0;i,j=1,2,\ldots ,S$). For any non‐negative parameter q≠1, we calculate an extent of homogeneity of pairwise distances as effective number of ordered pairs of OUs according to Hill (1973):

(1) Hq=∑i=1S∑j≠i=1Sfijq1/1−q,

whereas for q=1

(2) H1=limq→1Hq=exp−∑i=1S∑j≠i=1Sfijlogfij,

where $f_{\mathrm{ij}}=d_{\mathrm{ij}}/\sum_{i=1}^{S}\sum_{j\neq i=1}^{S}d_{\mathrm{ij}}$ is the proportional contribution of the ordered pair $\left(i,j\right)$ into the total distance between all pairs of OUs (we assume that $f_{\mathrm{ij}}\log f_{\mathrm{ij}}=0$ by definition. if $f_{\mathrm{ij}}=0$). Hq equals a hypothetical number of ordered equally distant pairs of different OUs ($d_{\mathrm{ij}}>0$, i≠j) that generate the same Hill number as the given set of $S^{2}-S$ pairs. This measure increases when variability in distances decreases, and range of Hq is between 0, if all $d_{\mathrm{ij}}=0$ (by definition), and its maximum $S^{2}-S$, when all $d_{\mathrm{ij}}\neq 0$ are equal for i≠j (*S* values $d_{\mathrm{ii}}=0$). Then diversity within the given set of OUs is obtained as solution of quadratic equation Dq2−Dq=Hq:

(3) Dq=1+1+4Hq2,

and expressed in terms of effective number of equally distant types of OUs (Scheiner et al., 2017). Values of Dq range from 1 to *S*, when all OUs are “identical” (all $d_{\mathrm{ij}}=0$) and all non‐identical OUs are equidistant ($d_{\mathrm{ij}}=\text{const}\neq 0$), respectively. Note, Dq gets smaller for larger q, and equal effect of all pairwise distances on the effective numbers is obtained just for q=1.

A kind of evenness of the OUs distribution is determined as

(4) Eq=Dq/S

with a range $\left[1/S,1\right]$. It is useful to transform this estimate onto the unit interval for comparison of sets with different numbers of OUs:

(4′) E′q=Dq−1/S−1

with a range $\left[0,1\right]$. So, diversity Dq increases with evenness and can be decomposed to the product of evenness and richness (number of OUs):

(5) Dq=Eq×Sor

(5′) Dq=1+E′q×S−1.

More accurately, Dq and EqE′q should be called diversity (effective number of equally distant populations [OUs]) and evenness of order *q*, respectively.

Diversity Dq reflects regularity of OUs distribution in a relevant space. It is determined by proportions $f_{\mathrm{ij}}$ and does not depend on actual distances $d_{\mathrm{ij}}$ between OUs in a sense that if all distances are subject to enlargement to the same extent, Dq remains unchanged since Hq does so. Thus, the effective number of equidistant OUs serves as an invariant of configuration of the given set in space (diversity perspective), while the degree to which OUs are similar to each other is not considered (dispersion perspective). Therefore, the diversity reveals an important component of biological variation, but not the complete structure of the metapopulation. Next, we will complement the diversity with dispersion perspective for a comprehensive description of variability within a set of OUs.

#### 2.3.2 Integration of diversity and dispersion

Theoretical aspects of dispersion and its relationship to diversity were broadly considered in Gregorius and Kosman (2017, 2018). To develop overall metrics of variation, we incorporate two of the most basic and tangible dispersion estimates. The first one is the Average Distance Within (ADW) a set of OUs

(6) $$\mathrm{ADW}=\sum_{i}^{S}\sum_{j}^{S}d_{\mathrm{ij}}/S^{2}$$

with a range from 0 to $\left(S-1\right)/S$, or its derivative ${\mathrm{ADW}}^{\prime }$ obtained by transformation of ADW onto the unit interval ($0\leq {\mathrm{ADW}}^{\prime }\leq 1$)

(6′) $${\mathrm{ADW}}^{\prime }=\frac{S}{S-1}\times \mathrm{ADW}=\frac{S}{S-1}\times \sum_{i}^{S}\sum_{j}^{S}d_{\mathrm{ij}}/S^{2}.$$

The second metric of dispersion is Kosman's assignment‐based measure KW (Kosman, 1996, 2014; Kosman & Leonard, 2007) that has a range $\left[0,1\right]$ and can be considered as generalization of the mathematical definition of the diameter of a set of scattered points.

Finally, we combine diversity (Dq) and dispersion (ADW or ${\mathrm{ADW}}^{\prime }$, and KW) estimates into integrated metrics of overall structural variation that we call the effective number of different populations (ENDP), or OUs:

(7) DADWq=1+Dq×ADW=1+S×Eq×ADW=1+S−1×Eq×ADW′,

(8) DKWq=1+S−1S×Dq×KW=1+S−1×Eq×KW

with a range from 1 to *S*. A general form of Equations (7) and (8) is

(9) DMq=1+S−1×Eq×M

for any dispersion metrics M with $\left[0,1\right]$ range. The immediate consequence is that even if diversity is maximal (Dq=S), that is, all OUs are equally distant (evenly distributed), the effective number of different OUs DMq decreases and approaches to 1 when OUs are closer to each other (dispersion decreases and tends to 0). According to Equation (9), the effective numbers of different OUs DMq can be represented as a decomposition of the three generally independent basic components: simple richness of a given set (*S*), evenness (Eq), and dispersion (M). The effective number of different OUs could be conceived as the number of equidistant OUs needed to obtain the same dispersion and variability in pairwise distances as those observed in the given set of OUs (where OUs may not be equally distant).

The suggested approaches to estimating variation can be thought of as reducing the actual number of OUs (richness) in two steps. Analyzing regularity of OUs distribution, richness (*S*) decreases to the effective number of distinct equidistant OUs (Dq) due to deviations from a perfect evenness. Then, considering a magnitude of similarity between OUs (dispersion) results in further richness decline from Dq to the effective number of different OUs (DMq for dispersion M). Thus, combining both the diversity and dispersion perspectives, overall variation of a set of OUs is expressed in terms of reduction of its simple estimate (richness) to perhaps the most exhaustive one—the effective number of different units. The effective numbers of different and equidistant units are equal only in two extreme cases: for a set consisting of one unit (trivial situation), and when all units are maximally distant.

To make a comparison of structural variation of sets with different numbers of OUs, relative estimates of the effective numbers (EN, 1≤EN≤S) are useful and reached by the linear transformation of EN onto the unit interval

(10) $$n\mathrm{EN}=\left(\mathrm{EN}-1\right)/\left(S-1\right).$$

nEN increases with increasing variation EN and can be considered the metric of structural differentiation of OUs. The relative effective number of equally distant OUs (nD) is obtained for EN=Dq from Equation (10), that is nDq=E′q is evenness from Equation (4′), while the relative effective number of different OUs nDMq is attained with EN from the absolute estimate DMq (Equation 9). These relative estimates (nEN) range from 0 (no differentiation) to 1 (completely structured set of OUs) when the corresponding effective number equals 1 and S, respectively. Both the metrics EN and nEN of variation among populations are obtained totally independent of variability within the populations because the latter was not even involved in generation these metrics of differentiation. This independence is reached using conceptually different approach comparing with those of Jost (2008, equations 8 and 10, p. 4021), which could be referred to as approaches based on the partitioning of diversity within and among OUs. Thus, the suggested metrics of structural differentiation nEN (Equation 10) are completely different from classical measures of differentiation. Importantly, some extent of statistical correlation between estimates of β‐ and α‐variation in any specific system is generally possible, although the metrics of β‐variation (EN) and its derivative (*nEN*) are mathematically independent of within‐sample α‐variation.

### 2.4 Data and differentiation among parasite populations

We tested the new metrics with a published dataset on genetic structure of a diploid trematode parasite *Atriophallophorus* spp. (Feijen et al., 2022). *Atriophallophorus* has a snail‐bird life cycle. It reproduces sexually in the bird definitive host. The adult worms are hermaphrodites but evidence supports outcrossing as main mode of reproduction (Feijen, 2020). The parasite reproduces asexually in the snail intermediate host. Feijen et al. (2022) reported a phylogeographic analysis of the most common *Atriophallophorus* species, *A. winterbourni*, but the study also revealed a previously unknown sister species coexisting with *A. winterbourni* (Feijen et al., 2022). This putative species remains undescribed. The study covered a wide geographic range (South Island of New Zealand) and applied both nuclear and mitochondrial markers in a detailed phylogeographic analysis of the studied populations. Here, we use these data to ask what the ENDP is when calculated with the new metrics we present. We first test how the new method performs when we apply it to samples representing the two main species. In our analyses, we mainly refer to figures 2 and 3, and figure S4 (Feijen et al., 2022). We use the same data that they analyzed for species delimitation among *Atriophallophorus* spp. (table S4.1 in Feijen et al., 2022), except that we excluded individuals with missing data and small samples (*N* < 10), which reduced the number of lakes with sufficient sample size from 15 to 10 lakes. We then limit the analysis to the most common species *A. winterbourni* and contrast effective numbers of equally distant populations (Dq) to ENDP [DMq]. Only polymorphic SNP loci were used in the analyses.

We estimated the variation among these parasite populations as follows:

1. We calculated the dissimilarity between the SNP genotypes (δ) according to equation (3) and the corresponding algorithm on p. 421 in Kosman and Leonard (2005) or equation (6) in Kosman and Jokela (2019). In the case of missing data, the corresponding loci were ignored for each pair, and a dissimilarity value was obtained on the reduced number of loci with available data for both individuals in the pair.
2. We computed the average‐based and assignment‐based distances using the δ‐dissimilarity (${\mathrm{DAD}}_{\delta }$ and ${\mathrm{KB}}_{\delta }$, respectively) between all pairs of populations.
3. We calculated the effective number of equally distant populations (diversity D1) according to Equations (2) and (3) for distances $d={\mathrm{DAD}}_{\delta }$ and $d={\mathrm{KB}}_{\delta }$, and q=1. Then the diversity‐based estimates of differentiation (nEN) were obtained for EN=D1 from Equation (10).
4. We calculated the dispersion of the parasite populations (${\mathrm{ADW}}_{{\mathrm{DAD}}_{\delta }}$ and ${\mathrm{ADW}}_{{\mathrm{KB}}_{\delta }}$) using Equation (6) (ADW based on distances $d={\mathrm{DAD}}_{\delta }$ and $d={\mathrm{KB}}_{\delta }$).
5. We calculated the ENDP [structural variation DADW1] according to Equation (7) for q=1 for the corresponding pairs of diversity D1 and dispersion ADW estimated with distances $d={\mathrm{DAD}}_{\delta }$ and $d={\mathrm{KB}}_{\delta }$ Then the corresponding assessments of structural differentiation (nEN) were obtained according to Equation (10) with EN=DADW1.

### 2.5 Effect of sample size and number of loci on the proposed metrics

We performed an analysis of robustness of the ENDP estimates to variation in sample size and variation in number of marker loci for genotyping. We analyzed resampled datasets where sample size or number of loci were reduced up to 50%. For each sample size/number of loci combination, we calculated the metrics from 10 replicate random draws without replacement from the original data. The full dataset consisted of 212 genotypes at 24 polymorphic diploid loci of the original sample representing 6 haplotype groups of the two main species *A. winterbourni* and *Atriophallophorus* sp. (Feijen et al., 2022).

## 3 RESULTS

### 3.1 Application of effective numbers of populations to mixed populations of cryptic species

Based on the species delimitation analysis, Feijen et al. (2022) concluded that at least two species of *Atriophallophorus* parasites were found in the studied populations. We calculated that the ENDP [DADWDAD1,DADWKB1] in the set of samples grouped by the six major mitochondrial haplotype groups was 1.40 when based on the distance of average differences (${\mathrm{DAD}}_{\delta }$) and 2.05 for the assignment‐based genetic distance $({\mathrm{KB}}_{\delta }$) (Table 1). While the difference between these metrics is 32%, here the assignment‐based distance seems to match the expectation of at least two species particularly well and average‐based distance seems to underestimate the number of inferred OUs.

**TABLE 1:** Variability among the trematode *Atriophallophorus* spp. collections.

Type of variation	Variation parameters	“cryptic” species/populations identified based on mt‐haplotype groups (Feijen et al., 2022)		*Atriophallophorus* populations (natural lakes)
		52 genotypes 24 loci 6 hapl. groups	212 genotypes 24 loci 6 hapl. groups	306 genotypes 24 loci 10 lakes
Effective number of equally distant populations (Diversity)	DDAD1 ^a^	5.544	5.445	9.783
	DKB1	5.914	5.911	9.990
Dispersion	${\mathrm{ADW}}_{\mathrm{DAD}}$ ^b^	0.071	0.058	0.015
	$\mathrm{ADW}\prime_{\mathrm{DAD}}$ ^b^	0.085	0.070	0.017
	${\mathrm{ADW}}_{\mathrm{KB}}$	0.178	0.169	0.171
	$\mathrm{ADW}\prime_{\mathrm{KB}}$	0.217	0.203	0.190
Evenness	EDAD1 ^c^	0.924	0.908	0.978
	EKB1	0.986	0.985	0.999
	E′DAD1=nDDAD1 ^c^	0.909	0.889	0.976
	EKB′1=nDKB1	0.983	0.985	0.999
ENDP, effective number of different populations (structural variation)	DADWDAD1 ^d^	1.396	1.316	1.146
	DADWKB1	2.053	1.999	2.698
Extent of differentiation	nDADWDAD1 ^e^	0.079	0.063	0.016
	nDADWKB1	0.211	0.200	0.189

^a^ Effective number (Equation 3).

^b^ Dispersion (Equations 6 and 6′; Kosman, 1996; Kosman & Leonard, 2007).

^c^ Evenness (Equations 4 and 4′).

^d^ Extent of differentiation—normalized ENDP (Equation 10).

^e^ Effective number (Equation 7).

**FIGURE 1:**
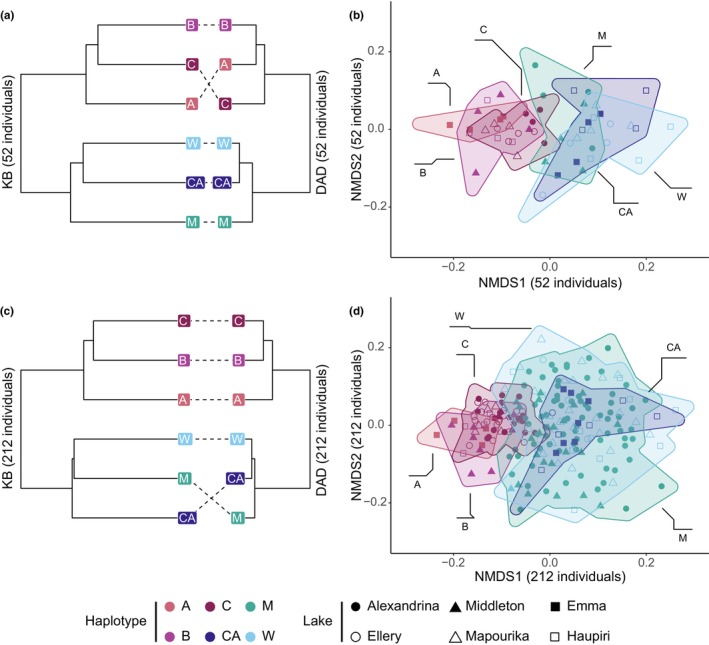
UPGMA dendrograms and NMDS plots of the two datasets (a, b: 52 individuals; c, d: 212 individuals). Panels (a) and (c) are based on pairwise KB (left) and DAD (right) distances between the six major mitochondrial haplotype groups reported in Feijen et al. (2022). Note that DAD topology in (a) is congruent with the tree shown in figure 2c in Feijen et al. (2022), while the top clade (haplotype groups B, C, A) show a different structure obtained with the *DAD* and *KB* distances in (a) and (c). Panels (b) and (d) show NMDS plots calculated based on pairwise distances between individuals. The haplotype group for each sample is indicated in the label.

As the calculation of these metrics does not demand as many computational resources as the Bayes Factor Delimitation models that Feijen et al. (2022) used, we were able to expand the analysis to the larger dataset that was used in the regression analysis in Feijen et al. (2022). Our results are very similar to the results reported by Feijen et al. (Figure 1; Table 1). Interestingly, the ENDP was not affected by the sample size (Table 1). This indicates that these metrics are robust to variation in sample size assuming the samples still represent the different OUs (here, haplotype groups).

Our results illustrate that the ENDP captures the underlying genetic structure in the *Atriophallophorus* clade (Figure 1). Although the species is sexual, it seems that in this case the association‐based KB distance was more strongly in agreement with previous analyses than distance of average differences (DAD). This may be due to low gene flow between the species emphasizing the differences between the species that appear as strong linkage (association between loci) when haplotype groups are compared. Note also that the effective number of equally distant populations, which reflects the diversity, was close to maximum defined by the six haplotype groups (Table 1). Interestingly, when diversity was calculated based on average (DAD) or association‐based (KB) distance the estimates only differed by 6% (Table 1). Analysis of number of equally distant populations does not capture the cryptic species structure in the clade, probably because it treats all haplotype groups independently of the magnitude of differences between them. In this case, using the additional information from dispersion was therefore essential to describe the previously inferred structural variation among the haplotype groups.

### 3.2 Application of effective number of populations to geographically separate populations of single species, *Atriophallophorus winterbourni*

Feijen et al. (2022) presented genetic pairwise $F_{\mathrm{ST}}$ and structure analyses for 15 lake populations of *A. winterbourni*. Their first discovery was that the nuclear marker‐based estimates for population structure were much less than mitochondrial marker‐based estimates. Their main conclusion was that in the past the populations were likely separated in glacial refugia in the north and south of the Island and that the present population differentiation in nuclear and mitochondrial markers is maintained due to low level of cross‐alpine migration. Average nuclear $F_{\mathrm{ST}}$ was low, and together with analysis of migration patterns using isolation by distance tests and marginal approximation of structured coalescence (phylogeographic analysis based on mitochondrial markers applying Mascot 2.1.2. in BEAST 2.6.5. [see details in Feijen et al. (2022)]), the conclusion was that even if the mitochondrial $F_{\mathrm{ST}}$ estimates were high, there is a considerable nuclear geneflow among all populations at present.

**FIGURE 2:**
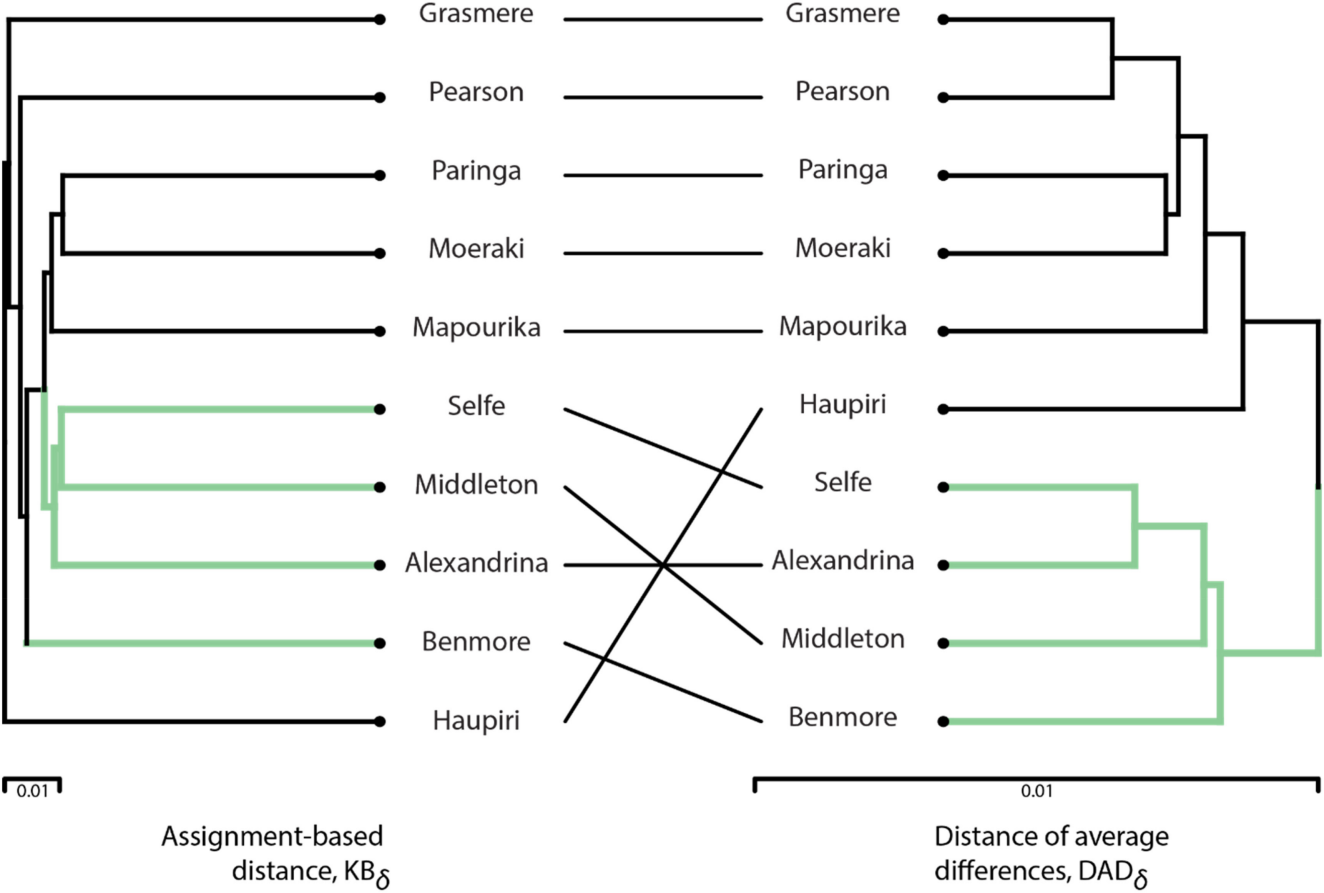
UPGMA trees of *Atriophallophorus winterbourni* populations from 10 lakes on the South Island of New Zealand. Data are the same as presented in table S4.1 of Feijen et al. (2022), with the exception that the lakes with small samples (less than 10 individuals) were excluded from the analyses. The colors of the branches correspond to two main clusters identified in the Structure analysis presented in Feijen et al. (2022, figure 3d). Effective numbers of different populations based on the DAD and KB distances are 1.15 and 2.70, respectively.

Our analysis using the DAD distance suggested that the ENDP in these data is 1.15 supporting the view that there may have been two distinct glacial refugia, but the nuclear marker‐based differentiation among the population is currently weak. However, using the association‐based *KB* distance the ENDP was 2.70 (Table 1). Figure 2 illustrates differences in relationships among the populations between the two estimates. In this case analysis, based on the distance of average differences *DAD* reflects the expected structural variation better than the association‐based KB distance. This may be expected as the data represent large outbred sexual populations that are in HW equilibrium showing no signal of linkage disequilibrium (Feijen et al., 2022).

### 3.3 Effect of sample size and number of loci on the proposed metrics

Estimates of four metrics of β‐variation obtained for randomly selected genotypes of 6 haplotype groups of the two main species *A. winterbourni* and and *A. winterbourne* are shown in Table 2. All estimates were calculated for 10 replicates in each case of 184, 148 and 112 genotypes subsampled from the 212 genotypes at 24 polymorphic diploid loci in the original sample. In addition, all estimates were calculated for 10 replicates in each case of a reduced number of 20, 16 and 12 loci for all 212 genotypes (Table 3). Values of the effective number of equally distant populations (diversity DKB1) changed in a very narrow range. On the other hand, variability of values of the three other metrics [dispersion ${\mathrm{ADW}}_{\mathrm{KB}}$, effective number of different populations DADWKB1 and extent of differentiation nDADWKB1] was much larger, and considerably increased with decreasing number of genotypes or loci as reflected by the coefficient of relative range: $\text{rrange}=\frac{\max-\min}{\max}$. Importantly, reducing the number of loci used for genotyping had a much stronger effect on variation of estimates of those three metrics than diminishing of sample sizes to the same extent. For example, values of rrange for ENDP [DADWKB1] increased above 2.6 times from 0.011 to 0.023 and 0.029 when the sample sizes of genotypes at 24 loci decreased from 184 to 148 and 112, respectively (Table 2). Similar tendency was in the case of nearly the same extent of reduction of the number of loci used for genotyping from 20 to 16 and 12 ones for 212 genotypes with an increase of above 2.7 times from 0.052 to 0.112 and 0.141, respectively (Table 3). However, the absolute values of variation (rrange) were about 5 times greater for the reduction in the number of loci (0.052 vs. 0.011, 0.112 vs. 0.023, and 0.141 vs. 0.029).

**TABLE 2:** Effect of sample size on metrics based on 10 replicates in each separate case.

Sample size	Loci	Metric	Ref. val.	Mean	SD^a^	Min	Max	rr‐range
184	24	ADW_KB_ ^b^	0.169	0.169	0.001	0.167	0.171	0.022
148	24	ADW_KB_	0.169	0.171	0.002	0.168	0.176	0.047
112	24	ADW_KB_	0.169	0.176	0.003	0.172	0.182	0.055
184	24	^1^ *D*_KB_ ^c^	5.910	5.914	0.002	5.911	5.917	0.001
148	24	^1^ *D*_KB_	5.910	5.919	0.008	5.900	5.933	0.006
112	24	^1^ *D*_KB_	5.910	5.923	0.009	5.903	5.937	0.006
184	24	^1^ *D*(ADW_KB_)^d^	1.999	2.000	0.008	1.989	2.012	0.011
148	24	^1^ *D*(ADW_KB_)	1.999	2.010	0.013	1.994	2.042	0.023
112	24	^1^ *D*(ADW_KB_)	1.999	2.040	0.017	2.018	2.079	0.029
184	24	^1^ *nD*(ADW_KB_)^e^	0.200	0.200	0.001	0.198	0.202	0.022
148	24	^1^ *nD*(ADW_KB_)	0.200	0.202	0.003	0.199	0.208	0.046
112	24	^1^ *nD*(ADW_KB_)	0.200	0.208	0.003	0.204	0.216	0.057

*Note*: Reference value (Ref. val.) is calculated for the full data of 212 genotypes.

^a^ Standard deviation.

^b^ Dispersion (Equation 6).

^c^ Diversity—effective number (Equation 3).

^d^ ENDP—effective number of different populations (Equation 7).

^e^ Extent of differentiation—normalized ENDP (Equation 10).

**TABLE 3:** Effect of number of loci on metrics based on 10 replicates in each separate case.

Sample size	Loci	Metric	Ref. val.	Mean	SD	Min	Max	rr‐range
212	20	ADW_KB_ ^b^	0.169	0.168	0.006	0.159	0.179	0.108
212	16	ADW_KB_	0.169	0.167	0.013	0.143	0.184	0.223
212	12	ADW_KB_	0.169	0.162	0.018	0.139	0.184	0.246
212	20	^1^ *D*_KB_ ^c^	5.910	5.899	0.010	5.887	5.921	0.006
212	16	^1^ *D*_KB_	5.910	5.881	0.026	5.839	5.919	0.014
212	12	^1^ *D*_KB_	5.910	5.862	0.027	5.808	5.912	0.018
212	20	^1^ *D*(ADW_KB_)^d^	1.999	1.990	0.035	1.944	2.051	0.052
212	16	^1^ *D*(ADW_KB_)	1.999	1.980	0.073	1.843	2.076	0.112
212	12	^1^ *D*(ADW_KB_)	1.999	1.947	0.103	1.820	2.118	0.141
212	20	^1^ *nD*(ADW_KB_)	0.200	0.198	0.007	0.189	0.210	0.102
212	16	^1^ *nD*(ADW_KB_)^e^	0.200	0.196	0.014	0.169	0.215	0.216
212	12	^1^ *nD*(ADW_KB_)	0.200	0.189	0.021	0.164	0.224	0.267

*Note*: Reference value (Ref. val.) is calculated for all 24 loci.

^a^ Standard deviation.

^b^ Dispersion (Equation 6).

^c^ Diversity—effective number (Equation 3).

^d^ ENDP—effective number of different populations (Equation 7).

^e^ Extent of differentiation—normalized ENDP (Equation 10).

Despite the substantial increase in the variability of estimates of dispersion, ENDP and extent of differentiation for 10 replicates with the trimmed number of genotypes and loci (2.6 and 2.7 times, respectively), the estimates themselves did not deviate considerably (up to 5% in terms of the replicate averages; Tables 2 and 3) from those obtained for the original data of 212 genotypes determined at 24 loci. However, it looks like a trend that the estimates slightly increased/decreased with the reduced number of genotypes/loci (e.g. for the extent of differentiation nDADWKB1, changes were from the original estimate 0.200 to 0.208 and 0.189 ones for 112 genotypes and 12 loci, respectively; Tables 2 and 3).

Maximum difference of a point estimate of the effective number of different populations for each separate replicate for the reduced numbers of genotypes (184, 148 and 112) or loci (20, 16 and 12) from the ENDP value for the original data (212 genotypes, 24 loci) did not exceed 4% for 112 genotypes at 24 loci and 9% for 212 genotypes at 12 loci. Worthwhile to mention that the ENDP [DADWKB1] estimates very strongly correlated with the dispersion (${\mathrm{ADW}}_{\mathrm{KB}}$) estimates (Pearson correlation coefficients exceeded 0.99).

## 4 DISCUSSION

Assessing genetic structure of populations requires that the chosen measures reflect the biological processes that affect local genetic variability and divergence among populations (Bohonak, 1999). Relevant processes shaping population genetic structure are well understood but capturing these processes to a single metric is difficult. For example, species mating system has consequences for the expected genetic variability of populations (Holsinger, 1992; Rieseberg & Burke, 2001), variation in population size affects the strength of genetic drift (Wang et al., 2016), and local adaptation may promote divergence of genes under selection (Yeaman & Whitlock, 2011). Metapopulations consist of local populations of different sizes, which may be connected by highly asymmetric geneflow (Harrison & Hastings, 1996; Morrissey & de Kerckhove, 2009). Recently evolved mating barriers may also lead to cryptic species structure that is yet unnoticed and further complicates the analysis of population genetic structure (Baker et al., 1995). Ideally, the chosen metric would be robust in the sense that there is no unrecognizable bias by specific biological processes or possible sampling errors. It would be very valuable if the metrics recorded would guide the inclusion and exclusion of alternative hypotheses to explain the observed patterns. It is unlikely that a single metric can capture all aspects of population structure, processes defining divergence of populations and methodological caveats that handicap our conclusions. Inference from several alternative metrics might allow concluding how the populations are structured, which processes are relevant and how the analyses can be refined to address specific follow‐up questions.

We aimed to show how beta variation among populations can be estimated independently of alpha variation within populations, to evaluate how metrics incorporating both the diversity (based on Hill numbers) and dispersion facets of variation can be used as beta variation estimates, and how they are best constructed to evaluate population genetic data from natural populations that differ in the processes that shape the population genetic structure. We focused on evaluating both diversity and dispersion emphasizing that both are important. The second aspect that we examined is the difference between average (DAD) and association‐based (KB) distance measures (Kosman & Leonard, 2007) when deriving effective numbers estimates. We showed that estimates of the ENDP based on the DAD distance are well suited for situations where studied OUs have low compatibility barriers generating association due to assortative mating (or fertility) patterns. If compatibility barriers (i.e., cryptic species) exist, then the KB distance used in calculating the ENDP capture the structural variation better.

We argue that the analysis of population genetic structure, genetic variability of populations and assessment of the conservation value of local populations would benefit from inclusion of both the diversity and dispersion aspect of structural variation when estimating genetic relationships of populations in a metapopulation (beta variation). We use examples from population genetics, but these same approaches can be utilized in study of biological communities using functional traits (Kosman et al., 2019; Scheiner et al., 2017). We believe that in this sense the recognition of diversity and dispersion perspective to variation is integrative and common to both genetics and ecology. It would be important to examine how such integration is best achieved and if there is a link between genetic and functional diversity, or genetic and functional dispersion. Here, we recognize the debate on the link between biodiversity and ecosystem function (de Laplante & Picasso, 2011; Grime, 1997). Maybe the anomalous results from the tests of this central hypothesis are actually due to lack of consideration of diversity and dispersion aspects of the taken measures. Are the used measures of diversity also capturing the dispersion of taxa that would best map on dispersion of ecosystem function? In other words, the metrics that measure dispersion (or metrics that combine both dispersion and diversity) might be closer to the objectives for testing the biodiversity‐ecosystem function hypothesis.

Our main interest was to ask how we best characterize structural variation in populations using population genetic markers. The classical approach in population genetics relies on a kind of apportionment (not differentiation!) measures (like $F_{\mathrm{ST}}$ and its relatives) that strictly deal with the diversity aspect of variation and are blind to dispersion. This does not seem a limitation when considering only one locus and assuming that all alleles are equally dissimilar. However, the limitations of the classical approach become real when one considers markers where the extent of similarity between different alleles at one locus may vary (e.g., microsatellites, Kosman & Jokela, 2019). At present, most genetic data consist of multilocus genotypes (e.g., any sequence of any kind). When examining such data, it is very easy to agree that not all genotypes are equally dissimilar; therefore, an analysis using information on variation in dissimilarity to support conclusions on structural variation of populations may be a useful addition. Using dissimilarity is implicit in coalescence models of evolution where evaluation of the shortest approach to ancestral type requires understanding of evolutionary distances of the derived types (Rosenberg & Nordborg, 2002). Evident power of coalescence‐based models is one of the reasons why we argue that also studies on structural variation of populations (population genetic structure/diversity) would greatly benefit from incorporation of the dispersion component into measuring of overall variation.

Another known shortcoming of applying the classical (apportionment) metrics to measuring differentiation among populations is the dependence of those metrics on variation within the populations (this is why they do not assess the differentiation) (Gregorius, 2014; Jost, 2008). The great advantage of using numbers equivalents to estimate variation within (alpha) and among units (beta) is that those estimates are independent (Jost, 2007). However, even the modified metrics developed for measuring differentiation (e.g., Jost's *D*) still depend on diversity within populations (e.g., counterintuitively Jost's *D* cannot reach its maximum value 1 even if two populations do not share any alleles and one of them is not fixed). The approach we advocate here (combining diversity and dispersion) to derive differentiation measures based on effective numbers of different OUs, provides efficient and tangible tools for analyzing relationships among populations, and allows comparisons across studies.

Our two examples illustrate how the effective numbers approach can be used in ecological genetics evaluating structural variation in natural populations. We emphasize the difference between assessments of the effective numbers of different OUs with average‐based and association‐based distance measures between the OUs. In some cases, where populations are large, outcrossing and not under strong selection or drift, metrics based on the distance of average differences are capturing the processes affecting structural variation among populations. This was the situation in our second example where geographically widespread species was inferred to have been divided into two major regions that had somewhat less geneflow between regions than within regions. In our first example, what was long assumed a single species in fact consisted of coexisting cryptic species that were morphologically similar but evolutionarily diverged (Feijen et al., 2022). Such cases are very demanding to discover with data that are collected to test hypotheses assuming a single species. Here, the proxy we used to construct evolutionary prior groups was the mitochondrial haplotype memberships. Finding such a prior grouping factor requires collection of additional data and processes such as incomplete lineage sorting may complicate matters further (Maddison & Knowles, 2006; Pedraza‐Marrón et al., 2019). For this case, we showed that association‐based ENDP captured the assumed cryptic species structure and could have been used to motivate further species delimitation studies with high confidence. Of course, here we have the advantage of hindsight as such analyses were already done (Feijen et al., 2022).

The analyses we present require that it is possible to have prior assumptions of OUs. We believe that collecting data with assumed a prior structure in mind is a much more productive approach than assuming no structure. Everything in biology speaks for assuming memberships of groups for observed individuals even if everything in statistics is based on constructing null models for assuming such groups/structures do not exist. For example, membership in the population can be assumed by spatial location, or by mitochondrial haplotype identity, as we show in our examples. Both spatial priors and haplotype identities can cross species boundaries, but they might still be useful starting points for structural analysis. Here, our first example relied on using priors based on haplotype groups, and the second relied on population membership. We believe that the power of using the suggested approach is that one can reduce the priors to the most likely number of different (genetically, functionally etc.) groups among the OUs in question thus providing important information about the structure in the data based on the corresponding estimate of effective number of different OUs. This is a philosophically different approach than asking the data (blindly) how many groups emerge when some clustering algorithm is applied. We think it is rare not to have a good candidate for prior grouping. Most data are collected assuming population membership. Therefore, asking about the effective number is a logical thing to do when analyzing the data. Most data are assigned to more populations than in fact are there since for most species the migration patterns and effective geneflow are not known partly due to the lack of conceptually sound methods of population delineation. This is an issue that is like the inference we receive from population size (number of individuals) and effective population size (number of individuals contributing to the next generation). We see value in assigning population memberships a priori and validating that count post hoc with effective numbers metrics and suggest this should be part of our routine beta diversity estimates when conducting studies on biodiversity, genetic diversity or functional diversity of populations.

To have a flavor of what future studies could reveal, we showed that preliminary analysis with simulated data demonstrated that number of loci used for genotyping had a much stronger effect on variation of point ENDP estimates than the sample size. Nevertheless, the estimates of ENDP seem to be rather robust because manipulations with numbers of genotypes and loci (up to 50% reduction) led to the deviation up to 4% and 9%, respectively, from the ENDP value obtained for the original data. Such deviations seem comparable with possible experimental errors (including genotyping) in similar studies.

## AUTHOR CONTRIBUTIONS

**Evsey Kosman:** Conceptualization (lead); formal analysis (lead); methodology (lead); software (lead); writing – original draft (equal); writing – review and editing (equal). **Frida Feijen:** Investigation (equal); validation (equal); visualization (equal); writing – original draft (supporting); writing – review and editing (supporting). **Jukka Jokela:** Conceptualization (equal); data curation (lead); formal analysis (equal); funding acquisition (lead); investigation (equal); methodology (equal); visualization (lead); writing – original draft (equal); writing – review and editing (equal).

## FUNDING INFORMATION

This study was supported by Swiss National Science foundation (grant #310030_207589 to JJ). Collection of the data presented in the paper was supported by a grant from the Swiss National Science foundation (grant # 31003A_166667 to JJ).

## CONFLICT OF INTEREST STATEMENT

All authors declare that they have no conflicts of interest.

## Data Availability

Data used in this study are available at https://datadryad.org/stash/dataset/doi:10.5061/dryad.2ngf1vhnw. User‐friendly software LOCUS and FDAT (Functional Diversity Analysis Tools) can be downloaded at https://en‐lifesci.tau.ac.il/profile/kosman. The software needs a programming environment of the Microsoft.NET Framework, which is an integral Windows component.
